# Commentary: Growth of Global Health Spending Share in Low and Middle Income Countries

**DOI:** 10.3389/fpubh.2017.00052

**Published:** 2017-03-14

**Authors:** Ana V. Pejcic

**Affiliations:** ^1^Faculty of Medical Sciences, University of Kragujevac, Kragujevac, Serbia

**Keywords:** global health, health expenditure, medical spending, low-income countries, middle-income countries

The paper by Jakovljevic and Getzen highlighted the fact that low- and middle-income countries have been grabbing an ever larger share of global health spending over the last couple of decades (1). Share of global health spending of low- and middle-income countries as of 1995 expressed in million current PPP international $US grew from 26.1% in 1995 to 39.7% in 2013 (1). These countries are led by nations of BRICS (Brazil, Russia, India, China, and South Africa), followed by Next-11 nations (Bangladesh, Egypt, Indonesia, Iran, Mexico, Nigeria, Pakistan, the Philippines, Republic of Korea (South Korea), Turkey, and Vietnam) with a joint contribution to the global total health expenditure several times below the one of BRICS (1–5). Low- and middle-income countries, which represent an immense range of health system contexts, are likely to have more significant contribution in the global health-care market in the future as it is estimated that per-capita health spending will increase annually by 2.4, 3.0, and 3.4% in low-, lower-middle-, and upper-middle-income countries by 2040, respectively (1, 6). For high-income countries this rate is estimated at 2.7% (6).

One interesting question can be raised. What is happening with population health outcomes in low- and middle-income countries as health expenditure is increasing? The authors mentioned that “substantial gains in overall welfare are reflected in the expansion of health insurance coverage and diversity of medical services provided” (1). Some other aspects would also be valuable for discussion.

First, determining the impact of health expenditure on health outcomes is a challenging and complex issue as health outcomes are determined by a vast number of socioeconomic and environmental factors (7–9). Solely increasing public health expenditure, may not significantly affect health outcomes if its efficiency is inadequate (8, 9). It has been suggested that, on average, inefficiency of allocating health expenditures in emerging and developing economies is highest in Africa, while Western Hemisphere and Asian economies are relatively more efficient, with significant variations within the aforementioned regions (8). One systematic review has shown that private health-care system sectors in low- and middle-income countries appear to have lower efficiency compared to public sector as a result of weak regulation, higher costs of drugs, improper incentives for unnecessary testing, and treatment, but that, on the other hand, public sector tends to be less responsive to patients and susceptible to the lack of availability of supplies (10). Higher public health expenditure is generally associated with better health outcomes, but still there are substantial differences within the emerging and developing economies groups (8). The relationship between public health expenditure and health-adjusted life expectancy, as well as immunization rates, is generally found to be positive and significant, whereas it is negative and significant with mortality rates (8). For example, favorable effect of higher public health expenditure on mortality under 5 years is significantly larger for low- and middle-income countries (11). However, this might not be applicable to all low- and middle-income settings. In some African countries, like Nigeria, increasing public health expenditure alone, without properly addressing issue of corruption, is not enough to lead to improvement in population health status (12).

Health-care quality improvement is very important for improving population health outcomes (13). However, it has been shown that increasing health-care expenditure does not necessarily reflect increasing quality of delivered health care (14, 15). The evidence from BRICS nations confirms that sole increase in public health expenditure cannot assure better health outcomes unless the quality of delivered health care is substantially improved (9). Even in the United States, where per-capita spending on health care is estimated to be 50–200% greater than in other developed countries, this does not yield much better health outcomes compared to other OECD countries (16) and higher spending is not highly correlated with the quality of care, as price of the same service may vary and expensive new therapies may be adopted without good evidence that they improve patient outcomes (17).

In 2012, International Journal for Quality in Health Care dedicated a special issue to address status of health-care quality improvement research in low- and middle-income countries with many papers that highlighted that “much remains to be studied and understood to optimally promote quality improvement” (18). Data on quality of health-care services in low- and middle-income countries are scarce, probably due to the past emphasis on health-care coverage rather than the quality of provided care and insufficient validation of the existing quality measures (19). Quality assessment in terms of infrastructure and staffing, technical quality, and patients’ experiences was not done consistently in low- and middle-income countries, thus comparing of measurements made in different settings is difficult (19). A systematic review based on limited data from comparative studies conducted in low- and middle-income countries suggested that the quality of private and public ambulatory care is similarly low in terms of infrastructure, clinical competence, and practice for both types of providers, although private sector tends to perform better in drug availability and aspects of delivery of care, such as responsiveness and effort (20).

Increasing burden of rising incidence of non-communicable diseases and accelerated population aging in low- and middle-income countries will pose a major problem for national policy makers (21–29). As Jakovljevic and Getzen pointed out, achievement of universal health coverage, types, and costs of services covered by basic insurance package will certainly remain the major imperatives for national policy makers of these countries (1). Governments will also need a comprehensive approach in order to develop and implement effective strategies to ensure adequate efficiency of forecast increase in health spending along with improving quality of care. Policy lessons from high-income countries may be useful, but they might not transfer well to all low- and middle-income countries’ settings due to the key context differences regarding widespread poverty and relative weakness of political and social institutions (15). In order to develop successful approaches, countries should take into consideration their own specific circumstances after careful evaluation and prioritization of underlying problems.

## Author Contributions

AP has designed, drafted, and finalized the manuscript.

## Conflict of Interest Statement

The author declares that the research was conducted in the absence of any commercial or financial relationships that could be construed as a potential conflict of interest.
